# The dark side of beauty: an in-depth analysis of the health hazards and toxicological impact of synthetic cosmetics and personal care products

**DOI:** 10.3389/fpubh.2024.1439027

**Published:** 2024-08-26

**Authors:** Abdullah M. Alnuqaydan

**Affiliations:** Department of Basic Health Sciences, College of Applied Medical Sciences, Qassim University, Buraidah, Saudi Arabia

**Keywords:** cosmetics, toxicity, mixtures, regulatory measures, risk assessment, health hazard

## Abstract

Over the past three decades, the popularity of cosmetic and personal care products has skyrocketed, largely driven by social media influence and the propagation of unrealistic beauty standards, especially among younger demographics. These products, promising enhanced appearance and self-esteem, have become integral to contemporary society. However, users of synthetic, chemical-based cosmetics are exposed to significantly higher risks than those opting for natural alternatives. The use of synthetic products has been associated with a variety of chronic diseases, including cancer, respiratory conditions, neurological disorders, and endocrine disruption. This review explores the toxicological impact of beauty and personal care products on human health, highlighting the dangers posed by various chemicals, the rise of natural ingredients, the intricate effects of chemical mixtures, the advent of nanotechnology in cosmetics, and the urgent need for robust regulatory measures to ensure safety. The paper emphasizes the necessity for thorough safety assessments, ethical ingredient sourcing, consumer education, and collaboration between governments, regulatory bodies, manufacturers, and consumers. As we delve into the latest discoveries and emerging trends in beauty product regulation and safety, it is clear that the protection of public health and well-being is a critical concern in this ever-evolving field.

## 1 Introduction

The application of cosmetics and personal care products has permeated every aspect of daily life for people all over the world in today’s social media driven image-conscious society. A staggering 90% of individuals use some form of personal care product daily. Personal care market revenue is expected to reach US $205.50 billion in 2023 (1). These products not only pledge enhancements in attractiveness but also tout the ability to boost self-confidence and foster overall well-being. These goods make the claims that they will improve attractiveness, increase self-confidence, and promote well-being. The risks posed by the harmful substances used in these products are masked beneath the attraction of beautiful skin and glossy hair. Recent studies have revealed the grave effects that utilizing such items has on human health, raising concerns regarding their safety and highlighting the requirement for strict regulation (2).

An abundance of items, ranging from cosmetics and hairdressing to fragrances, have flooded the market as the personal care and beauty industry has experienced exponential expansion. While the assurance of beauty enhancement has attracted many customers, considerable concern has been raised by 78% of consumers about the potential risks related to these products (3). The phrase “toxic beauty” has become popular, denoting the potentially dangerous effects that some substances may have on people’s health whether used topically or accidentally swallowed (4). Many cosmetic and personal care items contain a variety of chemicals that could be harmful to people’s health. Of particular concern, phthalates, found in plastics and fragrances, have been linked to developmental and reproductive challenges, affecting approximately 10% of the population (5). Moreover, the interaction between naturally sourced and synthetic chemicals can potentiate their toxicity, affecting a notable 30% of consumers who experience adverse reactions (6). Because of their propensity to impair hormone control and other endocrine functions, the preservative parabens, which are often employed, have drawn criticism. Phthalates, which are present in plastics and fragrances, have been linked to problems with development and reproduction (7).

There has been a shift toward formulas that feature botanical and naturally derived substances as a result of the rising desire for “clean” and “natural” cosmetic products. Even “natural” products, though, might have hazardous ingredients. The lack of appropriate examination of these chemicals is due to the misunderstanding that organic components are always safe. Additionally, some compounds that come from natural sources could come into contact with synthetic chemicals to increase their potential toxicity (8). Revenue margins and marketability are frequently given higher priority by businesses than thorough safety analysis. In fact, a staggering 65% of companies prioritize profit over meticulous safety evaluation, and a mere 15% of products undergo rigorous pre-market carcinogenicity testing (9). The problem is further complicated by the absence of established testing procedures and precise rules for evaluating ingredients. Customers are thus forced to negotiate a minefield of items with perhaps hidden risks. Due to the lack of established testing protocols and detailed guidelines for evaluating ingredients, consumers frequently find themselves navigating a complex landscape of products that may carry hidden risks, while businesses frequently place a higher priority on profit margins and marketability than thorough safety analysis (10).

There is a growing need for stricter controls as people become more aware of the risks that personal care and cosmetic goods may pose. In order to strengthen industry monitoring, advocacy groups, academics, and worried consumers are putting pressure on governments and regulatory agencies. They emphasize the necessity of enhancing controls and ensuring the safety of these goods (11). In this literature review, we will provide insight into the adverse health effects of cosmetic products in general, focusing on what is known about their toxic and genotoxic effects on human health and discussing the current regulations of cosmetics.

## 2 Exploring the diverse landscape of beauty product consumption among different age groups

The use of personal care and cosmetic products has spread across all ages and gender divisions and has become a mainstay in modern culture. People that want to look better, feel better about themselves, and follow grooming standards love these goods. This business has a wide range of products that are made for both men and women as well as for kids, demonstrating its diversity (12). The extensive usage of these products across a range of demographic groups must be examined, and caution must be exercised regarding any potential hazardous effects that may come along with their use, as the requirement for these products continues to soar (13). According to Husøy et al., the EuroMix human biomonitoring BM study was conducted to examine the concentration of toxic chemicals such as phenols and phthalates in urine samples, particularly exposure from food and personal care products (PCP). The results of this study showed that the proportion of phthalates, bisphenol A, and triclosan in urine samples was calculated to be approximately 88–100%. Face and hand creams, anti-aging creams, body wash and shower gel, toothpaste, and shaving cream were the main exposure to phthalates in men and women from PCP products. However, bisphenol S (i.e., it has two phenol functional groups on either side of its sulfonyl group), which is considered a highly toxic chemical similar to bisphenol A and is responsible for endocrine disruptors, carcinogens, and neuronal cell destruction, and its percentage in urine samples was 29% (14).

Using personal care and beauty items is an activity that both men and women engage in. It is not exclusive to any one gender. Due to historical trends and societal expectations, women’s goods often predominate the market; yet, there has been a noticeable increase in the accessibility and embracing of cosmetics made specifically for men (15). This change is the result of shifting ideas about masculinity and self-care, which has given rise to a wide variety of goods addressing the grooming requirements of males (16). As opposed to the more complex processes frequently associated with female beauty regimens, male-oriented goods frequently highlight simplicity, efficiency, and functionality. The market for male beauty goods now includes essential ingredients like salicylic acid for skin that tends to break out in pimples, anti-aging formulations, and specialty beard care products. The potential toxicity of some components remains a concern, despite any differences in focus, necessitating a review of the dangers for both sexes (17).

Products geared for men frequently highlight simplicity, effectiveness, and beyond adult demographics, children are also included in the use of cosmetics and personal care items (Infante et al., 2016). This expanding market satisfies parents’ need to guarantee the welfare and maintenance of their children. This market sector is distinguished by formulas that emphasize mildness and safety, from delicate shampoos and lotions for newborns to exciting and vibrant products targeting pre-teens (Liang, 2020). Children’s goods place a strong emphasis on the safety of their ingredients, frequently promoting “no-tears” formulae and hypoallergenic claims. Toxic and genotoxic concerns linked to certain substances should not be ignored in the effort to make products as gentle as possible (18). The substances used in goods for kids deserve close examination due to the fragile nature of developing skin and the possibility of long-term repercussions (19).

A thorough literature review was conducted to perform the meta-analysis using multiple academic databases including PubMed, Cochrane Library, Excerpta Medica Database, and Web of Science. The results were limited to studies that looked at the toxic effects of cosmetic and personal care products among cosmetologists and hairdressers, and this meta-analysis was published in 2016. For the purpose of the study, only studies that matched certain inclusion requirements, such as empirical research, peer-reviewed publications, and topical significance, were chosen. The findings of this meta-analysis give a summary of the papers that were examined, including a variety of harmful consequences. The consequences of some substances included in cosmetics and personal care products are not limited to, skin sensitization, but also induce endocrine disruption, reproductive disorders, infertility, and fetal death. A complex link between product formulations, exposure time, and health effects is revealed by the results of various investigations (20).

Several studies have revealed patterns and trends in adverse effects by synthesizing data from multiple studies. It becomes clear that several chemicals, including parabens and phthalates, which are frequently found in these cosmetics, have been linked to negative health impacts. Prolonged and repeated exposure, the synergistic effects of chemical combinations, and even possible nanoparticle penetration are all factors that can cause these hazardous effects (21). The ramifications of these discoveries for human health highlight the necessity of strict regulatory measures, openness in the sector, and educated customer choice. The analysis sheds light on the adverse effects of beauty and personal care products on human health, highlighting the urgent need for regulatory reform, educated consumer decisions, and continued research in the field. Securing human health becomes crucial in the changing cosmetics and personal care industry as people continue to rely on these products for aesthetic improvement (22).

Product safety assessment and assurance are under the purview of cosmetics and personal care manufacturers in the absence of strict regulatory control. Due to the lack of pre-market testing and the availability of constituent information, this self-regulation strategy enables businesses to launch items (23). Diverse groups use beauty as well as personal care goods, reflecting the common desire to display one’s best self to the outside world. This widespread practice, though, does not eliminate the necessity of taking individualized safety into account. The safety of the substances employed should be the primary consideration, even though formulations for various genders and age categories may differ (24). The widespread usage of cosmetics and personal care items among all genders and age groups demonstrates how popular they are now. The market is still evolving, influenced by cultural trends and personal tastes, from gender-inclusive formulas to items made for the youngest users. The risks these items may pose must not be overshadowed by their overwhelming attractiveness (25). The findings of this analysis make it clear that in order to protect consumers, there is an urgent need for tighter regulatory oversight of cosmetics and personal care items. The research has highlighted the value of educating and raising customer awareness of product constituents and hazards. Future studies should use standardized testing procedures in order to enable more accurate comparisons between various studies and products (26).

## 3 Ingredients in beauty and personal care products and their toxic and genotoxic effects on human health

### 3.1 Effects of chemicals ingredients to human health

The purpose of this paper is to provide a thorough understanding of this issue and to provide comprehensive knowledge based on recent research literature to reduce the use of restricted toxic chemicals worldwide. This review paper also offers valuable insights for nations struggling with the environmental impacts of cosmetics. According to statistics, cosmetic and skin care generated about $100 billion in 2022 internationally. From the data, it is evident that cosmetics and skin care products are consumed in a tremendous amount regularly due to unrealistic standards of beauty that are mostly triggered by social media these days (27). The human body is exposed to various toxic metals through inhalation, ingestion, and skin such as arsenic (As) (28), cadmium (Cd) (29), chromium (Cr) (30), lead (Pb) (31), and mercury (Hg) (23). Many toxic metals are added to skin care products to achieve the highest standards of beauty. Compounds with heavy metals when used in cosmetic products in excess of the recommended amount, can also cause various negative effects on the health of the human body. Metals contained in products can travel into the bloodstream through dermal absorption and cause dangerous effects on the human body (32). Genotoxic effects, in which specific compounds may cause DNA damage and mutation, are particularly concerning. Ingredients with genotoxic qualities, such as some pigments, UV filters, and even some organic compounds, increase the chance of developing skin cancer and other tumors. DNA repair activities are interfered with as part of the complex mechanisms of genotoxicity, which results in a buildup of mutations and cellular dysfunction (33). Beyond minor irritations, the effects of hazardous exposure from cosmetics and personal care items are extensive. Long-term contact with hazardous substances can cause a variety of health problems, from skin sensitivity and allergies to more serious problems like hormone disruption, reproductive problems, and even cancer (34). Numerous ingredients found in cosmetics and personal care items have been found to have the potential to cause cancer. The International Agency for Research on Cancer (IARC) has identified formaldehyde as a known human carcinogen. It is a frequent element in several nail paints and hair straightening products. Significant alarm has been generated by its potential to cause lung and nasal cancer (35). Unlike pharmaceutical products, cosmetic and skin care products have witnessed its share of moral questions over the past few years. This industry is under the cosh right now due to its environmental concerns, animal testing, and working conditions, but now the world is aware of environmental conditions and various strategies and developments have been made, especially for sustainability. Heavy metals and toxins have been found in industrial effluents resulting from skin care products. Many research articles have already highlighted the presence of heavy metals in samples collected from various wastewater treatment plants. For this purpose, continuous measures have to be taken to avoid emerging pollution of water resources (36). Toxic chemicals can be linked to cancer and transferred into the body through skin care products. However, in every state, no consumer product falls under less government oversight than cosmetic products. Although not all chemicals in cosmetic products are potentially dangerous, exposure to some toxic chemicals has been linked to chronic health conditions. According to a study, about 88 chemicals in more than 73,000 cosmetic products have been identified as toxins that cause headaches, dizziness, skin irritations, allergic reactions, and chronic diseases such as cancer and reproductive system disorders. The Toxic-Free Cosmetics Act, a new California law that will ban the use of 24 toxic ingredients in skin care products, comes amid worldwide concerns about the environmental impact of these toxic chemicals (37). The FDA (i.e., United States Food and Drug Administration) has banned certain chemical ingredients in cosmetics, the FDA has regulations and guidelines that specifically restrict the use of many ingredients in cosmetics including chloroform, mercury compounds, zirconium-containing complexes, and chlorofluorocarbon propellants (38). The present article aims to provide an up-to-date description of toxic ingredients that are harmful to human health and cause many biological disorders of human body organs such as 1,4-dioxane, acrylates, benzophenone, coal tar, butylated hydroxyanisole, carbon black, ethanolamine compounds, hydroquinone, heavy metals, methylisothiazolinone and methylchloroisothiazolinone, parabens, resorcinol, retinol, titanium dioxide, and many more.

### 3.2 1,4-dioxane

Attractiveness and intimate care products have become essential elements of contemporary living thanks to their tempting promises of greater attractiveness and self-esteem. The detrimental consequences that some of the substances found in these goods might have on human health, however, are hidden beneath the beautiful surface (39). This discussion digs into the complex area of toxicological issues surrounding these items, looking at the ways in which harmful substances might manifest their effects and emphasizing the demand for strict regulation measures (40). 1,4-dioxane is a heterocyclic ether organic compound, often used as an industrial solvent. It is an undesirable by-product of the ethoxylation process (i.e., the process of reacting ethylene oxide (EO) with other chemicals to make them less harsh) in the manufacturing of cosmetics. The International Agency for Research on Cancer (IARC) has identified and classified 1,4-dioxane as a potential human carcinogen. A substance’s capacity to cause cancer is referred to as its carcinogenicity. Carcinogens are substances, including chemicals, natural phenomena, and biological contaminants, that can start or encourage the growth of cancerous cells in an organism. It is a toxic ingredient that is not directly involved in the manufacturing process but has the potential to cause cancer and is found mainly in products such as shampoos and liquid hand and body soaps (41). According to Zhou (41), 1,4-dioxane in cosmetics can be measured by using a technique called gas chromatography combined with tandem mass spectrometry (i.e., a detection technique that ionizes and fragments molecules). This method has been widely used in the pharmaceutical and biomedical fields and has the advantage that the mass-to-charge ratio of the ions can be easily measured and the structure as well as the chemical properties of the unknown molecule can be determined with high certainty. In this methodology, a total of 82 different categories of cosmetic products such as baby hair and bath products were included in the study. As a result, 47 of the 82 products contained 1,4-dioxane with an average amount of 1.54 micrograms/gram (μg/g) in children’s skincare products (42).

### 3.3 Acrylates

The nail Cosmetic industry is escalating worldwide and generated worldwide revenue of US$ 12 billion in 2022. Acrylates also known as prop-2-enoates are stemmed from acrylic acid and are mostly used in the cosmetic nail industry. Mostly beauticians and artists are in the firing line for getting work-related issues and resulting occupational dermatosis such as hand dermatitis (i.e., a common acute or eczematous disorder) (43). People are exposed to this chemical by inhalation or skin contact. Exposure to the toxic components of nail products has resulted in many adverse effects on the human body, which have been reported worldwide. Recent studies have linked exposure to acrylates to the risks of cancer, reproductive organ toxicity, and skin irritation (44). The usage of a wide range of items promising improved appearances and increased self-confidence is closely related to the goal of cosmetics and personal care. However, these items’ attractiveness masks a worrisome truth: they contain chemicals that may cause cancer. Understanding the dangers posed by specific substances, the scientific basis for their cancerous potential, and the need for stricter regulatory measures to protect human health all depend on the examination of this unsettling dimension. The existence of potentially cancer-causing substances in cosmetics and personal care items highlights the significance of strict regulatory policies and thorough safety reviews. The current regulatory environment, nevertheless, frequently falls short of appropriately addressing these worries. Since many of these items do not go through thorough pre-market carcinogenicity testing, it is largely up to the producers to ensure safety (45).

### 3.4 Oxybenzone (benzophenone-3)

Benzophenone (Ph_2_CO) is a white organic compound found in most skin care products. Depletion of the ozone layer causes an increase in average ultraviolet (UV) radiation on earth and, in turn, increases the use of sunscreen and personal care products worldwide. Children are more likely to be affected by these UV rays and need different skin care products on the face and body regularly (46). Oxybenzone (benzophenone-3) is an organic compound that is recognized as an environmental pollutant and is used worldwide to help reduce the harmful effects of UV radiation. Oxybenzone is an important ingredient found not only in sunscreens but also in many personal care products such as shampoos, body lotions, and lip balms (47). There is growing concern that exposure to oxybenzone chemicals may be contributing to the increased incidence of endocrine system dysfunction in humans and other organisms. According to Ruszkiewicz et al. (47), Oxybenzone has been reported in several animal studies to disrupt the hypothalamic-pituitary-gonadal (HPG) system by causing severe downregulation of hormones. Measurable amounts of oxybenzone have been detected in human urine, and it is supposed that this is due to sunscreen use (48). It may be associated with increased susceptibility to organ system toxicity, skin irritation, and cancer risk (49). Consumer education is essential in getting manufacturers and regulatory agencies to put safety first. Consumers can help identify and remove carcinogenic compounds from the market by demanding transparency in ingredient disclosure and backing independent research on product safety (50).

### 3.5 Heavy metals

In the last few decades, cosmetics, pharmaceuticals, textile industry, and many other industries have faced many questions as these industries pollute the environment more, most of their environmental concerns are related to the heavy toxic metals used, along with the fact that they are environmentally persistence, bioaccumulative and toxic (PBT) (51). In European countries, the use of heavy metals in cosmetics is regulated by the European Regulation on Cosmetic Products (2009), for example, Annex II of Regulation (EC) 1223/2009 does not allow the use of heavy metals even at a low concentration except for mercury (Hg) compounds that are allowed for use as preservatives, which are listed in Annex V (52). As technology advances, heavy metals in cosmetics and personal care products have exceeded the limits set by regulators around the world. Skin contact is the primary source of human exposure to heavy metals in cosmetics. The effects of applying cosmetics on the face and body with heavy metals such as lead (Pb), arsenic (As), cadmium (Cd), and mercury (Hg) have now gradually attracted the attention of concerned people in policy-making departments (53). Heavy metals are very dangerous and unsafe for human health even at very low doses and duration of exposure. The purpose of adding heavy metals to cosmetics and personal care products is to act as an additive to enhance their shine, beauty, glow, and brightness. These heavy metals are mostly found in these products such as sunscreens, moisturizers, whitening toothpaste, nail polish, concealer, lipstick, and eyeliner (30). Lead (Pb) is one of the important heavy toxic metals because of its harmful effects on human, and animal life. According to Ara et al. (30), acute lead poisoning causes many symptoms in the human body and affects the human body badly. A human blood lead levels rise from 25 to 60 μg/dL, neuropsychological problems occur with symptoms of motor nerve dysfunction, loss of concentration, and headaches (54).

Lead poisoning also affects pregnant women, with elevated blood lead levels causing untimely birth of babies. Research has revealed that apart from men and women, children are also easily affected by lead poisoning, children can easily ingest and breathe lead-contaminated products (55). Research has shown that high levels of heavy metals affect cellular functions of cells and when these metal ions interact with DNA and cause DNA damage, resulting in organ system dysfunction and cancer. Heavy metals have the ability to alter the genetic makeup of cells and cause mutations that can cause uncontrolled cell proliferation and tumor growth, often linked to their ability to cause cancer. The normal processes of DNA repair and replication can be interfered with by carcinogens, which ultimately results in genetic instability (56).

Growing epidemiologic evidence and increased access to frequently compiled data on heavy metal concentrations and health facts and figures allow us to generate robust estimates in routine health impact evaluations. According to a report, in 2015, 35% of coronary heart disease (CHD) or coronary artery disease and approximately 42% of strokes worldwide were attributed to exposure to toxic chemicals (49). Experimental studies have found a positive relationship between exposure to heavy metals and some changes in the nervous system, such as oxidative stress, shortness of breath, memory, vision, and/or cognitive impairment, autonomic component imbalance, muscle cramps, and headache. However, additional research is needed to better explore the relationship between heavy metal exposure and chronic health problems (57).

### 3.6 Coal tar

Dyes are mainly used in cosmetic products as a dye agent to increase the attractiveness of the product to consumers. Dyes can be obtained from natural or synthetic sources. Coal-tar dyes, also recognized as synthetic dyes, are frequently used in hair treatment procedures in hair color products to prevent hair from looking dull and lackluster (58). Hair coloring is one of the most important parts of beautification that men and women have done since the dawn of man. The chemical composition of coal-tar dyes is quite complex, and phenolic compounds account for most of the components. Coal tar contains aromatic compounds, phenolic compounds, and heterocyclic nitrogen and oxygen compounds. Aromatic compounds are usually obtained from the distillation of petroleum (59). The detrimental effects of coal tar on human health have been frequently reported by numerous epidemiological studies worldwide, and it has been estimated that it can cause cancer and organ system toxicity worldwide. Besides cosmetic industries, other industries also use coal tar in its products such as food, textiles, and personal care products. Direct exposure to this toxic chemical via the skin can cause skin irritation and neurological system dysfunction (60). According to Lee et al. (60), coal tar dyes can cause visual impairment when applied to eyebrows and eyelid lashes. Many countries, such as the USA, have completely banned and outlawed the use of coal tar dyes in cosmetics and personal care products, especially around the eyes and face, due to the possibility of eye irritation or vision impairment (61).

### 3.7 Parabens and phthalates

Numerous substances found in cosmetic and personal care products have been linked, at the molecular level, to toxicological disturbances. Common preservatives like parabens have endocrine-disrupting qualities that can disrupt hormone homeostasis. Phthalates, a group of chemicals commonly used in fragrances, plastics, and various personal care products, have gained increasing attention due to their potential health and environmental impacts. These chemicals are primarily employed to enhance the flexibility and durability of plastics, including those found in packaging materials and medical devices (62). However, their presence in cosmetics and fragrances is a growing concern, especially when it comes to exposure through dermal contact and inhalation. Phthalates disrupt normal biological processes through their ability to mimic hormones in the body, primarily estrogen (63). As endocrine disruptors, they can bind to hormone receptors, interfering with the body’s endocrine system and potentially leading to hormonal imbalances. By doing so, they may impact various physiological functions, such as reproductive development, thyroid function, and metabolic regulation. This interference with hormone signaling can have broad-ranging effects, contributing to health issues like developmental and reproductive abnormalities (64). Moreover, phthalates can influence gene expression patterns, further complicating their effects on the body’s intricate regulatory processes. The exact mechanisms through which they disrupt endocrine signaling are an active area of research, and ongoing studies are uncovering more about the intricate ways in which phthalates interfere with normal biological functions (65).

The impact of phthalates on aquatic ecosystems and wildlife is a growing area of concern, as these chemicals can affect aquatic organisms and disrupt the balance of ecosystems. Studies and regulatory efforts around phthalates are ongoing, with some regions imposing restrictions and bans on their use in specific products, particularly those aimed at children (66, 67). Another category of preservatives called formaldehyde releasers has the ability to promote protein cross-linking, which can result in oxidative damage and DNA damage. There are many literature studies focusing on the acute effects of parabens (68). Parabens are preservatives associated with adverse health outcomes, including endocrine disruption and skin cancer, and are widely used to inhibit microorganisms in the food and cosmetic industry worldwide. The chemical structure of parabens are esters of para-hydroxybenzoic acid with alkyl or aryl components (69). The European Union has enacted legislation to limit the use of parabens in makeup, beauty, or personal care products. Recently, in 2014, the European Union amended its laws and regulations and further lowered the concentration of parabens in cosmetics and baby products, for example, Regulation No. 1004/2014 has now further reduced the permitted concentration of certain parabens, including propylparaben (i.e., a propyl ester of phenol p-hydroxybenzoic acid) and butylparaben (i.e., a butyl ester of phenol p-hydroxybenzoic acid) (70). Exposure to parabens through skin care products is a big problem, affecting both men and women alike, mostly found in shampoos, conditioners, and lotions. A recent review of exposure to parabens and breast cancer by Hager et al., found that there is growing recognition that exposure to chemicals, such as parabens, may contribute to the development of breast cancer (71). Environmental exposure to toxic chemicals such as parabens due to their release into the environment in large quantities from cosmetic factories not only contributes to endocrine disruption but also causes developmental and reproductive toxicity.^1^

### 3.8 Hydroquinone

Hydroquinone, also known as benzene-1,4-diol or quinol, is an aromatic organic compound used as a skin-lightening agent in personal care products such as face and skin cleansers, and facial moisturizers. Hydroquinone concentration is also regulated by the European Regulation in cosmetic products, listed in Annex II, for example, Regulation No. 1223/2009 does not allow the use of hydroquinone in any cosmetic products, except in one case, in which 0.02% concentration of hydroquinone is permitted in artificial nail products, which is listed in Annexure III (72). Considerable past evidence shows that skin-lightening agents such as hydroquinone are a major factor in causing many diseases in the human body such as respiratory tract irritation, organ toxicity, and cancer. It also causes greater harm to vulnerable populations, such as children, women, and especially young adults worldwide (73).

### 3.9 Methylisothiazolinone, methylchloroisothiazolinone, butylated hydroxyanisole, and butylated hydroxytoluene

The compounds methylisothiazolinone and methylchloroisothiazolinone, which are white solid organic compounds, butylated hydroxyanisole which is a synthetic, waxy, solid petrochemical, and butylated hydroxytoluene which is a lipophilic organic compound, these are all types of preservatives that have been used for nearly a century in many industries for their antiseptic properties, including the cosmetics, food, and pharmaceutical industries (74, 75). The main purpose of using these kinds of preservatives in cosmetics is to prevent the adhesion, attachment, and reproduction of microorganisms to the product. Various types of preservatives in skincare and cosmetic products can cause many side effects. The use of artificial preservatives in cosmetic products can not only cause cancer but also cause developmental and reproductive and organ system toxicity (76). Research shows that preservatives have different effects on living organisms and that is why there is constant research to find out how deleterious they are and how to remove them from the global market. In a report published in 2015, Okereke et al., found that preservatives such as BHA can cause itching, irritation, redness, bumps, hives, and swelling in the skin. The use of BHA is prohibited by European regulations and IARC in skin care products including makeup, sunscreens, lip and hair products, fragrance, and creams (77). A research report by Ahmad TMK has revealed that the harmful effects of artificial preservatives can cause genetic toxicity (78). In order to better understand the potential health risks associated with cosmetics, Table 1 provides list of common harmful ingredients found in cosmetics, skincare, and personal care items. This table summarizes the chemical names of these ingredients, the types of products they are commonly found in, and the associated health risks.

**Table 1:** Common harmful ingredients in beauty products and associated health risks.

Chemical name	Common products	Health effects/hazards	References
**Preservatives**			
Methylisothiazolinone	Shampoos, lotions, conditioners	Skin irritation, contact dermatitis, allergies	(163)
Methylchloroisothiazolinone	Haircare products, soaps	Skin irritation, contact dermatitis, allergies	(164)
Butylated Hydroxyanisole (BHA)	Lip products, moisturizers	Skin irritation, endocrine disruption, carcinogenicity	(21)
Butylated Hydroxytoluene (BHT)	Skin creams, lipsticks	Skin irritation, endocrine disruption, carcinogenicity	(165)
Formaldehyde-releasing Preservatives	Hair products, nail polish	Skin irritation, carcinogenicity concerns	(166)
Imidazolidinyl Urea	Various cosmetics	Skin irritation, formaldehyde release	(167)
Diazolidinyl Urea	Skin creams, lotions	Skin irritation, formaldehyde release	(168)
Sunscreen Chemicals	Sunscreens	Skin allergies, hormone disruption (e.g., Oxybenzone)	(88)
Parabens (e.g., Methylparaben)	Various cosmetics	Hormone disruption, breast cancer concerns	(169)
Propylparaben	Lotions, deodorants	Skin irritation, hormone disruption, allergies	(170)
**Plasticizers**			
Phthalates (e.g., Dibutyl Phthalate)	Nail polish, fragrances	Hormone disruption, reproductive toxicity	(15)
Diethyl Phthalate	Fragrances	Hormone disruption, allergies	(171)
**Heavy metals**			
Lead (in certain color additives)	Lipsticks, hair dyes	Neurological damage, reproductive issues	(172)
Mercury (in some skin-lightening products)	Skin creams	Neurological damage, kidney and lung toxicity	(173)
**Coal tar dyes**			
Coal tar (found in some hair dyes)	Hair dyes	Carcinogenicity, skin irritation	(174)
**Fragrance**	Various cosmetics, perfumes	Allergies, skin irritation, respiratory issues	(171)
**Triclosan**	Antibacterial soaps, toothpaste	Hormone disruption, antibiotic resistance	(175)
**Talc**	Powders, eyeshadows, blushes	Lung problems (when inhaled as dust), ovarian cancer risk	(176)
**Mineral oils**	Lotions, creams	Skin irritation, clogged pores, long-term skin issues	(177)
**Ethanolamines (MEA, DEA, TEA)**	Shampoos, body washes	Skin irritation, allergies, carcinogenicity (DEA)	(124)
**Microplastics**	Exfoliating scrubs, toothpaste	Environmental harm, potential ingestion and absorption	(178)
**Nanoparticles**	Various cosmetics	Potential skin penetration, long-term health effects	(179)
**Hydroquinone**	Skin lightening products	Skin irritation, ochronosis (skin darkening)	(180)
**Oxybenzone**	Sunscreens	Hormone disruption, potential allergies	(181)
**Sodium Lauryl Sulfate (SLS)**	Shampoos, body washes	Skin and eye irritation, allergies	(182)
**Toluene**	Nail polish, hair dyes	Nervous system damage, developmental issues	(183)
**Resorcinol**	Hair dyes, acne treatments	Skin irritation, allergies, thyroid dysfunction	(184)
**Polyethylene Glycols (PEGs)**	Lotions, creams, cleansers	Skin irritation, allergies, potential contaminants	(185)
**Formaldehyde**	Nail products, hair straighteners	Skin and respiratory irritation, carcinogenicity	(186)
**Retinyl Palmitate (Vitamin A)**	Anti-aging creams	Skin irritation, photosensitivity, potential carcinogenicity	(187)
**Artificial fragrance chemicals**	Various cosmetics	Allergies, skin irritation, respiratory issues	(171)
**Ammonia**	Hair dyes, hair color products	Eye and lung irritation, potential allergies	(188)

This table provides a list of various harmful ingredients commonly found in beauty products.

## 4 Effects of nano ingredients to human health

Nano cosmetics, sometimes known as Nano cosmetics, include skincare, haircare, and other cosmetics that make use of nanotechnology. Nanotechnology is the process of altering materials at the atomic or molecular level to produce systems, gadgets, and structures with distinctive features. This frequently entails utilizing nanoparticles in cosmetics—extremely small particles with sizes typically between 1 and 100 nm—to carry substances deeper into the skin or enhance the product’s capabilities (79). Nanotechnology, with inherited properties in cosmetics and skin care products such as good chemical reactivity, improving UV protection, skin penetration, color, finish quality, anti-aging effect, and transparency, was introduced in the cosmetic industry about 30 years ago. In cosmetic products, ‘nanomaterial’ refers to insoluble or bio-persistent and intentionally formulated materials. It must have one or more external dimensions, or internal structures, on a scale of 1–100 nm (80). Compared to other micro technologies, this technology is growing rapidly due to the superior properties of nanomaterials, including good surface area, hydrophobicity, flexibility, and high bioactivity (81). According to Sanches et al., there are some studies that have highlighted that titanium dioxide nanoparticles TiO_2_ NPs can trigger genetic alterations, inflammation, and cell toxicity, and these effects can be exacerbated after exposure to ultraviolet A and B rays. The process of toxicity consists of oxidative stress (OS), which results in the formation of reactive oxygen species such as superoxide anion (O_2_^−^), hydrogen peroxide (H_2_O_2_), and hydroxyl radical (HO^−^) in various dermal cells (82).

Metal oxide nanoparticle applications are gaining popularity worldwide in beauty care products including sunscreens, and face powders, as UV filters or brightening agents such as zinc oxide (ZnO) and titanium oxide nanoparticles (TiO_2_ NPs). The use of metal oxide nanoparticles in cosmetics products is also regulated by the European Union Regulation (EUR). EUR has revised its rules and regulations regarding the use of titanium dioxide in personal care products in 2021. For example, Regulation No. 1223/2009 was amended in 2021 to allow the use of titanium dioxide in beauty care products, which is listed in Annex III (83). Titanium dioxide is permitted in powder form with a diameter of ≤10 μm at a maximum concentration of 1% and about 25% only in loose powder form for facial products. Dermal exposure to these nanoparticles can induce many health problems in the human body (84). In this section, the toxic effects of these metal oxide nanoparticles on human health are analyzed extensively. There are a few drawbacks when it comes to using nano products.

Safety Issues: The likelihood that nanoparticles will pierce the skin and enter the bloodstream is the main safety issue with Nano cosmetics. This’s potential long-term repercussions are not well understood.

Environmental Impact: Nanoparticle manufacturing and disposal may have an impact on the environment. These particles may wind up in water systems after being wiped off, where they can have an impact on aquatic life. Regulation Concerns Nanotechnology in cosmetics is a very new topic as of my most recent update, and regulations in many nations may not have kept up to address the particular difficulties and potential concerns involved with Nano cosmetics (85).

There is an increasing need for more stringent safety testing procedures and regulatory reforms within the cosmetics and personal care sector as scientific understanding of carcinogenicity develops. To reduce the dangers posed by carcinogenic chemicals, stricter pre-market evaluations, standardized testing procedures, and mandated reporting of adverse effects are crucial. To ensure that goods meant for personal care and aesthetic enhancement do not compromise human health, governments, regulatory bodies, producers, and consumers must work together. We can advance toward a future where beauty and personal care products actually contribute to well-being without compromising safety by combining scientific rigor, consumer education, and lobbying for enhanced regulation (86).

## 5 Natural-based ingredients effects to human health

Natural ingredients have been used for beauty purposes for many years and now they are gaining more attention due to the various toxic and genotoxic effects of various other synthetic ingredients on the human body. The addition of natural-based components has led to a transformational shift in the beauty and personal care industries toward goods that satisfy customers’ needs for authenticity and well-being (87). These elements, which are gifts from nature, resonate with consumers looking for an alternative to synthetic formulations because they convey a sense of harmony and purity. However, the effects of naturally derived chemicals go beyond simple perception, providing a range of advantages that demand respect and careful study (88). There are many products in the market that claim to be “natural products” but the consumer should pay attention to the ingredients label. Natural products contain a variety of ingredients including aloe vera, apple cider vinegar, avocado oil, argan oil, coconut oil, bentonite clay, cedarwood oil, hemp seed oil, hyaluronic acid, kojic acid, jojoba oil, L-ascorbic acid (vitamin C), lavender oil, licorice root extract, olive oil, rosemary extract, shea butter, sandalwood, tea tree oil, and sunflower seed oil (89).

Natural-based ingredients include a wide range of botanical extracts, plant-derived oils, and minerals. Each of these compounds has special qualities that can improve the skin, hair, and general well-being. These ingredients frequently include vitamins, antioxidants, and vital fatty acids that can help skin stay hydrated, fight against environmental stressors, and even encourage the creation of collagen, which helps skin look younger (90). Traditional treatments and holistic methods are frequently used as inspiration for the incorporation of natural-based substances in beauty and personal care products. These ancient customs acknowledge the innate relationship between nature and health and attribute healing abilities to plants and other natural resources (91). Products that appeal to individuals looking for a comprehensive approach to self-care are the result of the harmonic fusion of ancient wisdom and modern science. Beyond a person’s personal health, natural components also appeal to people who care about the environment and ethical issues. Products with ethically sourced natural components are popular with those trying to reduce their environmental impact as consumers embrace sustainability. Compared to their synthetic counterparts, the cultivation and extraction of these components frequently entail less resource-intensive techniques (92). Additionally, encouraging ethical sourcing techniques encourages respect for regional communities and growers, improving livelihoods while maintaining traditional knowledge. This is consistent with the principles of conscious consumerism, in which people make decisions that take into account factors other than their own interests, such as the needs of society and the environment (93).

The substantial adverse effects of naturally based ingredients on humans have been demonstrated but are generally not appreciated. However, many people are often not conscious of the reality that natural ingredients are a complex mixture of many chemical compounds and that these ingredients inappropriately usage may induce many toxic effects on the human body (94, 95). Most studies have shown that using natural ingredients such as lavender oil, lemon oil, and various essential oils can trigger many types of skin allergies such as eczema and hives. Some studies have also associated lavender and tea tree oil to hormone disruption in males (96). However, at present, many people have shown interest in buying natural products due to the increasing health risks associated with synthetic products. Moreover, much research-type literatures highlight the positive effects of using plant extracts that have antiseptic and antioxidant properties and are also useful in the treatment of various diseases at a low cost (97).

After observing the toxicity or genotoxicity of certain types of synthetic preservatives in cosmetic and personal care products, scientists are searching for natural preservatives that are not as harmful as synthetic ones. Recently, the use of natural ingredients in cosmetic products is in high demand (3). Herbal ingredients such as cinnamon are gaining attention for having good antimicrobial activity and potency and are not as harmful as synthetic parabens such as methylparaben. According to research, a test was conducted to evaluate the antimicrobial activity of cinnamon essential oil against various antimicrobial pathogens such as *candida albicans*, *staphylococcus aureus*, *rhodotorula glutinis*, *micrococcus agilis*, *saccharomyces cerevisiae*, and *bacillus*. This type of micro-organism has been found in a variety of women’s cosmetic products including lipsticks, lip glosses, whitening creams, anti-aging creams, eyeliners, foundation creams, body creams, powders, and mascaras. The techniques used in this study were thin-layer chromatography (TLC) and gas chromatography with mass spectrometry (GC–MS) (i.e., an analytical method that combines the properties of gas chromatography and mass spectrometry and evaluates various kinds of compounds in a sample). MIC values (i.e., the lowest concentration of antibiotic at which bacterial growth is completely inhibited) ranged from 2.9 to 4.8 mg/mL. The results indicated that gas chromatography with mass spectrometry technique was effective in identifying the chemical composition of the substances and indicated the presence of cinnamaldehyde and its MIC value was 4.3%, which is considered good. This shows that this substance is very effective against *candida albicans*, *staphylococcus aureus*, *rhodotorula glutinis*, *micrococcus agilus*, and *bacillus strains* (98–100). However, cosmetics based on natural ingredients can be less toxic, but not totally safe as being advertising by the cosmetic companies.

## 6 Mixture effects on human health

Chemistry and creativity come together in the intriguing world of cosmetics to produce potions that can transform. These cosmetics were created by carefully combining numerous chemicals and compounds, promising beautiful skin, lush hair, and appealing fragrances. However, underneath the surface glamor, there may be a more sinister aspect: the potential harm that these products could do to people’s health. Making cosmetics is an intricate procedure that requires the harmonic blending of many substances that have been selected for their unique capabilities. To ensure product stability, scientists and formulators carefully blend synthetic chemicals, extract natural ingredients, and add additives and preservatives as part of extensive research and development (100).

A beauty product’s component list can resemble a scientific catalog. While humectants like glycerin draw in and hold moisture, emollients like oils and butter provide hydration and a smooth texture. Thickeners keep the viscosity of the solution consistent while surfactants, which resemble small cleaning agents, remove dirt and oil. Colorants and fragrances heighten the sensory experience and engage our senses. The potential harm these items could do to human health is what’s worrying, though. Despite serving as a barrier of protection, our skin can absorb some of the chemicals in these items, allowing them to interact with the systems in our bodies. There are numerous effects (101).

Allergies and skin irritability are frequent adverse effects of numerous cosmetics. Fragrances, preservatives, and specific colorants are chemicals that can cause reactions ranging from minor redness and itching to severe allergic reactions. Endocrine disruptors are some of the substances found in cosmetics. These chemicals, such as parabens and phthalates, disrupt the body’s hormonal balance and may cause a number of health problems. Due to their associations with cancer, carcinogenic substances such as formaldehyde-releasing preservatives and coal tar dyes have generated concerns. Consistent exposure to such substances has unsettling long-term effects (102).

Additionally, it is impossible to overlook how cosmetics affect the environment. When these goods are wiped off, the chemicals they contain may enter water systems and have a negative impact on ecosystems and aquatic life. Another issue is the cumulative exposure to a wide range of chemicals from using numerous cosmetics on a daily basis. Over time, the cumulative effects of these exposures may cause health issues. The absence of regionally consistent regulations increases complexity (103). Globally, there are regional variations in the regulation of cosmetic additives, which gives leeway for potentially dangerous compounds to enter goods with little inspection. The job of consumers is to remedy these issues. It is essential to read labels and become knowledgeable about potentially dangerous components. Reduce the risk by picking items with fewer potentially dangerous chemicals and natural substitutes. Cumulative exposure can be reduced by keeping beauty procedures simple and using fewer products. Patch testing new goods prior to full application can also aid in detecting negative reactions early on. Promoting openness in the beauty sector is crucial. Positive change can be sparked by encouraging companies to fully disclose their ingredient lists and by supporting legislative initiatives that put the needs of consumers first (104). Exposures to chemical mixtures have reportedly produced unexpected effects. These effects include enhanced acute and chronic responses, low-level concentration response, and unexpected target organ attack Harold I (105). Studies show that when the human body is exposed to mixtures of chemicals that enhance levels and produce effects that are not expected from an individual chemical. The evaluation of the finished products to human health should be done as long as the individual chemicals. The effects can vary and un expected effects can be observed.

The potential carcinogenic effects of chemical mixtures in cosmetics raise substantial concerns regarding public health (106). This concern arises when seemingly innocuous chemicals, each individually possessing a relatively low carcinogenic potential, converge within a cosmetic formulation to create a mixture that exhibits an unexpectedly elevated risk of inducing cancer (107). A central mechanism that amplifies this risk involves synergistic effects, where the collective impact of these chemicals surpasses the mere summation of their isolated carcinogenic potentials (108). This synergy can lead to increased DNA damage and the uncontrolled proliferation of cancerous cells, intensifying the overall carcinogenicity of the mixture. Potentiation serves as another critical factor, where one chemical within the concoction can significantly augment the carcinogenicity of another without the converse occurring, ultimately making the entire mixture more perilous (109). Metabolic activation is yet another integral component of this complex interplay. Some of the chemicals inherent to cosmetics require metabolic processes within the human body to become carcinogenic. When combined, these chemicals can induce a mixture more prone to metabolic activation, elevating the risk of cancer development (110).

Cumulative exposure poses a considerable risk as well. Regular use of multiple cosmetics over protracted periods can result in repetitive exposure to relatively low levels of potentially carcinogenic compounds. Over time, these exposures accumulate and may contribute significantly to the development of cancer, making the long-term effects of such exposure particularly disconcerting. Furthermore, the presence of certain chemicals in cosmetics has the potential to disrupt the delicate balance of hormones within the body, a phenomenon closely associated with the initiation and progression of cancer (111). The existence of endocrine-disrupting chemicals in cosmetics compounds these concerns, as they may interact in complex ways with the body’s hormonal systems.

These multifaceted mechanisms collectively underscore the pressing need to scrutinize the safety of cosmetic formulations rigorously. Equally vital is the advocacy for greater transparency in ingredient labeling, empowering consumers to make informed choices about the products they use (112). As ongoing research continues to unveil the intricate nuances of chemical interactions within these mixtures, both regulatory agencies and consumers must maintain unwavering vigilance to mitigate the potential carcinogenic risks entailed by cosmetics. Safeguarding public health necessitates ongoing efforts to understand, regulate, and minimize the unforeseen dangers lurking beneath the allure of these products (113).

In conclusion, the chemistry of cosmetics is a sophisticated fusion of art and science. Although these products promise to improve, it is important to be aware of the potential health risks they pose. As consumers, we can strike a balance between improving our beauty and protecting our well-being by exercising educated judgment and standing up for safer substitutes. However, regulations should be made for the examination of finished products as well before releasing them into the market, not focusing only on the individual ingredients of the products. The mixture of ingredients (formula of the finished products) is what people actually exposing to. As mentioned earlier, chemicals in the mixture can change their mechanism in a way that we do not understand to become carcinogenic or more toxic to human health (105).

## 7 Regulation of cosmetics

A key component of assuring consumer safety and product effectiveness is product regulation. Government organizations provide rules and guidelines to supervise cosmetics globally, preventing false advertising and potential injury. Cosmetic items in the US are primarily regulated by the Food and Drug Administration (FDA), even though pre-market approval is not necessary. Instead, they enforce laws to guarantee quality control, proper labeling, and safety (113). The security of cosmetic chemicals is of the utmost importance. Due to potential dangers, the FDA keeps a list of chemicals that are forbidden and restricted. To guarantee that ingredients are safe for their intended use, manufacturers must conduct safety evaluations. This evaluation procedure considers variables like component concentration and possible interactions. Another crucial component of regulation is the precise labeling of cosmetics. Ingredients, usage instructions, and potential dangers must all be correctly listed on labels. A product can be labeled as a medicine and be subject to additional regulations if it makes drug-like claims about its abilities, such as the ability to repair wrinkles. To stop customers from using items that could not have the desired results or might even be harmful, it’s crucial to make this distinction (114).

Cosmetic production procedures must adhere to GMP guidelines to ensure they meet quality requirements. These recommendations address a variety of topics, such as sanitation, equipment upkeep, and quality control procedures. Manufacturers can maintain consistent product quality and safety by following GMPs (115). Color additives, which are used to improve the cosmetics’ aesthetic appeal, must adhere to certain rules. To avoid potential health dangers, the FDA keeps a list of permitted color additives, and their use limits are strictly controlled. Manufacturers are urged to voluntarily disclose any adverse events brought on by their products under terms of adverse event reporting. Through this reporting system, the FDA is able to keep track of the safety of cosmetics and spot any potential hazards that might not have been visible during the first testing (23).

Despite the FDA’s jurisdiction to examine production sites to verify compliance with rules, cosmetic producers are not required to register with the organization. This guarantees that facilities follow GMPs and uphold the necessary quality requirements. Collaborations and agreements on a global scale have an impact on cosmetic rules. The International Cooperation on Cosmetics Regulation (ICCR), for example, works to synchronize legislation in various nations. By harmonizing regulations, the cosmetics industry hopes to promote international commerce while upholding high standards for quality and safety (116). The current regulation of cosmetic regulated by FDAs that Under U.S. law, most likely in Asia as well and other countries cosmetic products and ingredients, other than color additives, companies do not need FDA approval before they go on the market. Cosmetics products must be safe for consumers when being used according to products label that must clarify all the information needed. However, it is not required for the cosmetic companies to share their products safety data with FDAs. Also, the administration has no authority to require companies to demonstrate the safety of beauty or personal care products before releasing them on the market. Instead, it is self-regulation by the companies to decide the safety of the products. The role of the FDAs will be response to the consumer request if they experience any effects of using such products. The FDAs agency does not test or examine products, unlike drugs or medicines that required examination and clinic trials and get approve from FDAs before use (Federal Food, Drug, and Cosmetic Act, section 602, c).

However, there are still issues with the regulatory environment. The quick influx of new cosmetics onto the market might put a burden on regulatory resources, possibly resulting in supervision gaps. Additionally, while new cosmetic compounds are continuously developed, regulatory actions may not keep up, allowing some substances to be used until evidence of danger materializes.

Europe is a dominant cosmetics exporter and a world leader in the cosmetics industry. The EU’s role in particular concerned with establishing a regulatory framework for market access, regulatory convergence, and international trade relations. The EU has developed a special database called Coslng^2^ which allows easy access to look into cosmetic substances. Cosmetics legislation developed by the EU involves; all the cosmetics products being registered in the cosmetic products notification portal (CPNP) before the product is placed in the EU market, ensuring that no animal testing must be done to evaluate cosmetic products’ safety,^3^ market surveillance at the national level is the responsibility of the EU countries, and special attention must be given to some special product due to higher potential risk of these products to consumer health.^4^ At EU, the Regulation (EC) N° 1223/2009^5^ is the main regulatory framework for cosmetic products. The regulation ensures cosmetic products’ safety. This regulation replaces the old Directive 76/768/EC.

The EU cosmetics legislation generally prohibits the use of CMR (carcinogenic, mutagenic, and toxic for reproduction) in cosmetic products (117). However, special provisions are placed in certain circumstances. According to the Cosmetics Regulation 1223/2009 Article 15, the CMR substances are divided into different categories such as 1A, 1B, or 2 and are banned from being used in cosmetics however certain provisions on the use of CMR in cosmetic production are allowed.^6^

## 8 Safety in cosmetic means

The term “safe” firstly denotes the thorough examination of components. Each ingredient should go through dermatological and occasionally ophthalmological testing before being included in a cosmetic composition to make sure it will not irritate skin or produce an allergic reaction or other long-term health issues. Many countries have specialized regulatory organizations, such as the Food and Drug Administration (FDA) in the United States, that keep lists of authorized chemicals based on their safety profile (118).

But “safe” encompasses more than just quick responses. Additionally, ingredients must be examined for any potential long-term side effects, including endocrine disruption, reproductive toxicity, and carcinogenicity. A product that has been determined to be safe should not have negative effects after repeated use (87). Additionally, “safe” in today’s context increasingly refers to morally upright. Sustainable ingredient sourcing and testing have become integral components of safety considerations (92). In essence, “safe in cosmetic means” emphasizes a comprehensive method of product creation, where user safety right now and longer-term ethical consequences are of utmost importance (119).

## 9 Safety evaluation of beauty and personal care products

Beauty product safety requires a thorough testing procedure that supports consumer well-being and guards against potential harm. Before putting cosmetics and beauty goods on the market, manufacturers go through a thorough series of safety evaluations to determine the items’ acceptability for general usage. These tests cover a wide range of variables that work together to ensure the efficacy of the products and the safety of individuals who use them. A thorough examination of the ingredients is essential to this process. Ingredients in cosmetics are thoroughly examined to determine their safety characteristics. This entails a careful evaluation of all potential dangers, including those involving skin irritability, allergic reactions, and long-term health impacts (120). To protect consumers, substances with recognized safety issues may be banned or excluded from cosmetic compositions. The assessment of exposure is a crucial component of the safety examination. This necessitates taking into account both the frequency of use and the quantity of each ingredient used in a product. By doing this, it is feasible to evaluate whether the product’s constituent levels remain within safe ranges even when used often and on a regular basis (121).

Risk assessment is just the beginning of the process; manufacturers also conduct toxicity research. To assess their potential to be harmful, certain compounds may need in-depth toxicological testing. These studies frequently involve extensive testing, such as evaluations of skin sensitivity, tests for ocular irritation, and even analyses of possible systemic toxicity. Another important consideration is how well products are labeled. Manufacturers make sure that the product label accurately includes all of the contents and properly alerts customers to any potential dangers. This guarantees that customers are knowledgeable and capable of making wise judgments on the things they use (122).

The distinction between pharmaceuticals and cosmetics, however, also plays a role in this safety review procedure. A product may be put through more extensive testing to confirm its safety and efficacy as a medicine if it makes claims that go beyond its aesthetic use, such as treating particular skin disorders. Although regulatory bodies like the FDA may examine the safety evaluations that manufacturers submit, it is ultimately the manufacturer’s obligation to ensure that their products are safe (123).

In addition to the traditional safety evaluation methods mentioned earlier, the field of cosmetic safety assessment has seen advancements in recent years, particularly in the use of *in silico* approaches (106, 124). These computational methods have provided valuable tools for evaluating the effects of cosmetics on consumer well-being. *In silico* predictive modeling is at the forefront of these innovations. It leverages computer-based modeling and simulation to predict the safety and efficacy of cosmetic ingredients. These methods involve the use of advanced algorithms and databases to analyze the chemical structures of ingredients and predict their potential interactions with biological systems. For example, predictive toxicology models can estimate the likelihood of skin irritation or allergic reactions by analyzing the chemical properties of ingredients and their known effects on skin (108). High-throughput screening is another noteworthy approach. It combines *in silico* methods with automated laboratory testing to rapidly evaluate a large number of cosmetic ingredients. Computational models are used to prioritize ingredients for experimental testing based on their predicted safety profiles. This approach helps identify potential hazards more efficiently, reducing the need for extensive animal testing.

Toxicogenomics is yet another technique making strides in the cosmetic safety assessment. It examines how cosmetic ingredients affect gene expression and molecular pathways within the body. By analyzing the genetic responses to various ingredients, researchers can gain insights into potential long-term health impacts and identify ingredients that may disrupt normal cellular functions (125). Structure-Activity Relationship (SAR) analysis plays a pivotal role too. It involves studying the relationship between the chemical structure of cosmetic ingredients and their biological activities. *In silico* SAR models can predict the potential for a chemical to bind to specific receptors or enzymes, providing insights into mechanisms of action and potential health effects (126).

Furthermore, the integration of big data and bioinformatics has become increasingly feasible through *in silico* methods. The collection and analysis of vast amounts of data related to cosmetic ingredients, including their chemical properties and safety profiles, aid in identifying potential safety concerns. Bioinformatics tools are used to mine this data for patterns and trends, further enhancing our understanding of ingredient safety (127). Regulatory agencies have recognized the value of *in silico* approaches in cosmetic safety assessments. They may require manufacturers to incorporate computational modeling and data analysis into their safety evaluations, especially for new or untested ingredients (128). Incorporating *in silico* approaches into the safety assessment of beauty and personal care products offers several advantages. It reduces the reliance on animal testing, accelerates the evaluation process, and provides a more comprehensive understanding of ingredient safety. However, it’s important to note that *in silico* methods are most effective when combined with traditional safety testing and regulatory oversight to ensure the highest level of consumer protection.

As cosmetic manufacturers continue to innovate and introduce new ingredients, *in silico* approaches will play an increasingly vital role in enhancing the safety evaluation process, contributing to the development of safer and more effective beauty and personal care products.

## 10 Risk assessments of cosmetics

The thorough process of assessing the dangers associated with cosmetic components is known as risk assessment, and it is based on data analysis. The protection of consumer health and safety depends entirely on this process. Let us examine the fundamental elements of risk assessment, supported by relevant data. The procedure entails a careful analysis of each component of the cosmetic, starting with the evaluation of ingredient safety. Since its founding in 1976, the independent Cosmetic Ingredient Review (CIR) Expert Panel, which is tasked with examining the safety of cosmetic ingredients, has painstakingly examined a wide range of more than 2,700 substances. This emphasizes the dedication to meticulous evaluation (129).

**Figure 1:**
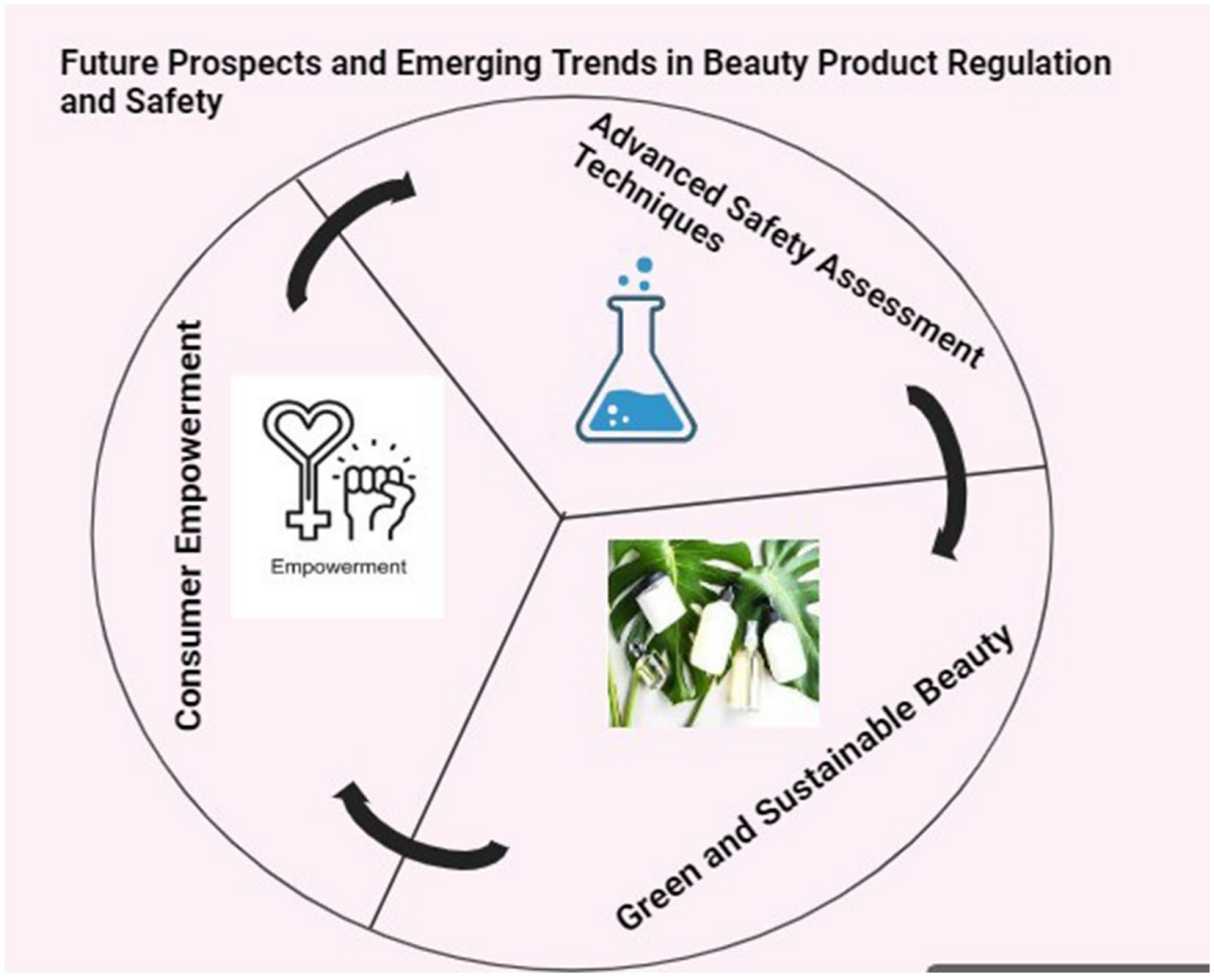
A holistic and multifaceted approach to safety testing in the cosmetics industry: ensuring consumer well-being and product quality through comprehensive evaluation and innovation in cosmetic formulations.

The dose-response relationship is a key component of risk evaluation. This method looks at how the dosage of a specific substance affects the body’s reaction. Skin sensitization testing, a method that involves applying several quantities of a chemical to determine the threshold at which an allergic reaction may potentially occur, is an actual illustration. In the process of assessing risks, exposure assessment is of utmost importance. Understanding the frequency and length of exposure to a specific cosmetic product is crucial since it has a substantial impact on the dangers that could be present. To assure the safety of consumer usage, the Scientific Committee on Consumer Safety (SCCS) of the European Commission, for instance, conducts exposure evaluations (117). This is demonstrated by the rigorous analysis of the components used in hair color (130). Figure 1 illustrates the different types of safety testing of cosmetic and skin care products across industries (131).

Additionally, the idea of cumulative exposure becomes a major worry. The cumulative exposure to phthalates from various cosmetic items is examined in a study that was published in the “Journal of Applied Toxicology.” The study emphasizes how important it is to consider the overall exposure brought on by using various products (132). It is also vital to highlight that cosmetic products’ ingredients as a single or mixture must be evaluated for safety. The final product must be free from any toxic effects. Within the scope of risk evaluations, vulnerable populations, such as children and pregnant women, receive special consideration. Due to their growing physiological systems, the American Academy of Paediatrics, for example, advises a careful approach to the use of cosmetics in young children. Risk assessments are essential to the development of testing techniques. *In vitro* assays are considered as an alternative approach to animal testing and as such sophisticated and cost-effective *in vitro* models are available to cosmetic industries for products testings. For example, *in vitro* test tube models are modern approaches that provide more accurate predictions of skin sensitization (133).

The use of different animals such as rabbits and rodents to assess the safety and efficacy of cosmetic products was considered a common practice. The mentioned animals were used for systemic toxicity evaluations, skin absorption, acute toxicity, mutagenicity, genotoxicity, carcinogenicity, reproductive toxicity, eye and skin irritation tests, etc. (134). However, in the recent past several countries issued mandatory regulations for alternative testing methods which resulted in discontinuing animal testings. Additional to that, the consumers also support cosmetic products which are cruelty free and not tested on animals (130, 135).

## 11 Future prospects and emerging trends in beauty product regulation and safety

**Figure 2:**
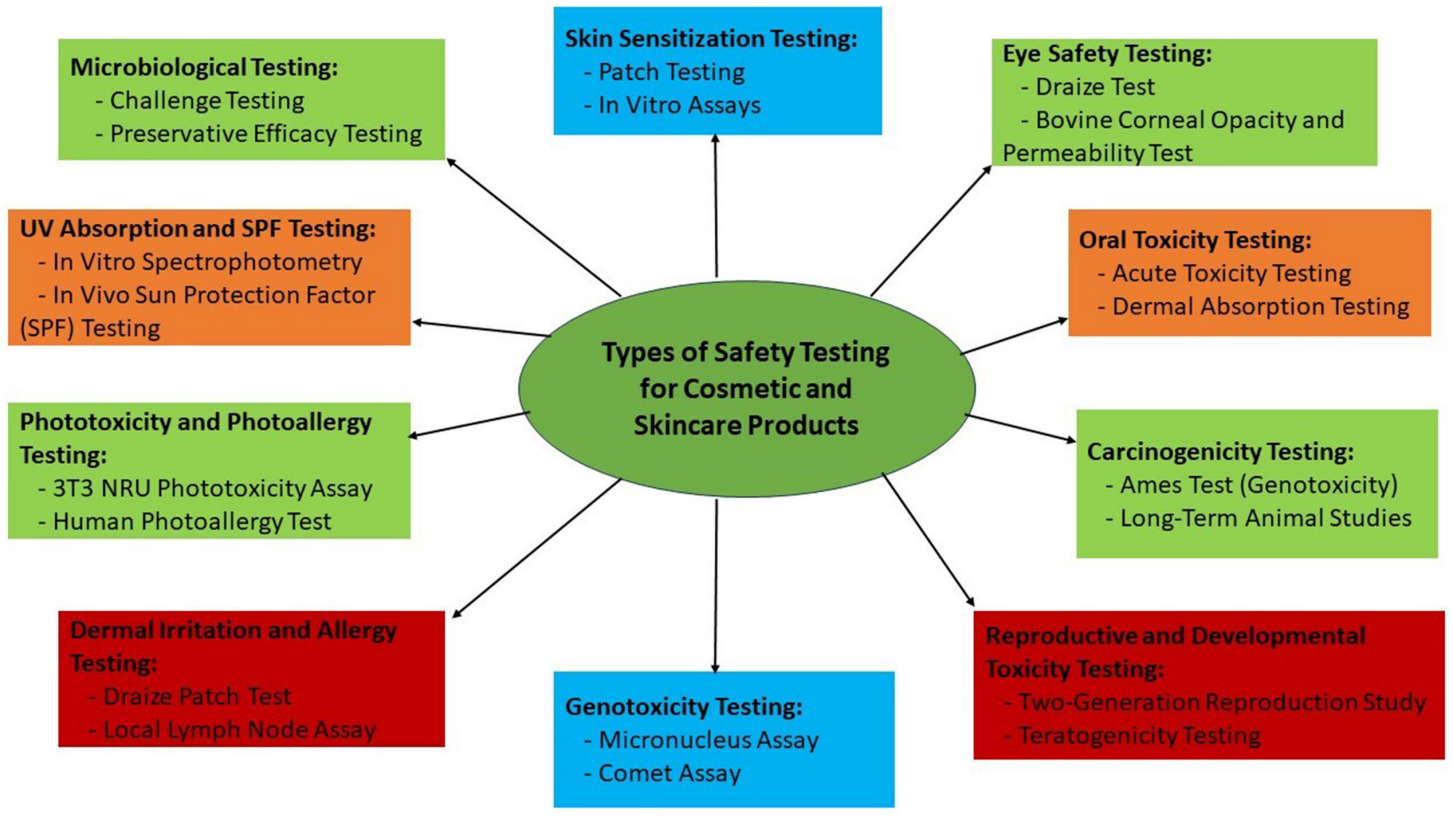
An illustrated exploration of future prospects and emerging trends in beauty product regulation and safety.

In the dynamic landscape of beauty product regulation, three interconnected elements drive industry evolution. Advanced safety assessment techniques embrace ethical alternatives to animal testing. Green and sustainable beauty practices prioritize eco-friendly materials and ethical sourcing. Consumer empowerment, fueled by information accessibility, steers trends toward clean, ethical choices. Together, these elements shape the beauty industry toward a future defined by safety, sustainability, and consumer-centric innovation (Figure 2).

### 11.1 Advanced safety assessment techniques

In recent years, significant advancements in safety assessment techniques for cosmetics have revolutionized the cosmetics industry. These innovations have paved the way for more accurate, efficient, and reliable methods to evaluate the safety of cosmetic ingredients and formulations.

One major breakthrough is the shift away from traditional animal testing toward *in vitro* testing and the use of 3D skin models. These advanced models replicate human skin structure and function, allowing for more precise evaluations of skin irritation, sensitization, and absorption (136). For instance, a study published in the Journal of Toxicology *in Vitro* (2022) demonstrated the effectiveness of a 3D human skin model in assessing the safety of various sunscreen formulations. The model accurately predicted skin compatibility, reducing the need for animal testing and providing more reliable safety assessments (137). The use of alternative skin models (ASM) for testing purposes attracted the attention of both industries and academia. The ASM development is now considered crucial in the field of dermatology as animal models studies are time consuming, costly, react differently compared to human skin, and issues of ethical concerns. The ASM like reconstructed skin, organ on a chip, *ex vivo* skin, and different computational models hold more promising applications than the animal models (138).

Advancements in computational toxicology have also played a pivotal role. Researchers have developed predictive models that estimate the safety of chemicals and their potential effects on human health (139). Machine learning algorithms and data-driven approaches can analyze vast datasets to identify potential hazards, making safety assessments quicker and more data-driven. A recent article in Computational Toxicology (2023) introduced a machine learning-based predictive model for evaluating the skin sensitization potential of cosmetic ingredients, achieving high accuracy in identifying potential allergens and accelerating safety evaluations in product development (140).

High-Throughput Screening (HTS) techniques have gained prominence in safety assessments, enabling the rapid testing of numerous compounds simultaneously (141). This approach enhances the efficiency of identifying potential safety concerns and expedites the development of safer cosmetic formulations. In a study published in the Journal of High-Throughput Screening (2021), HTS was used to assess the genotoxicity of cosmetic ingredients, showcasing the feasibility of evaluating a wide range of ingredients for DNA-damaging potential, a critical aspect of safety assessment (141).

Emerging technologies, such as “organs-on-chips,” offer a dynamic and physiologically relevant platform for safety evaluations. These microfluidic devices mimic the functions of specific organs, enabling the study of systemic effects and interactions of cosmetic ingredients (142). An article in the journal Nature Biomedical Engineering (2022) described the development of a liver-on-a-chip model for assessing the metabolism of cosmetic compounds, holding promise for understanding how the body processes and eliminates ingredients, contributing to safety assessments (143).

These advanced safety assessment techniques represent a significant leap forward in ensuring the safety and quality of cosmetics, while also addressing ethical concerns related to animal testing. They are shaping the future of beauty product regulation by providing more accurate, efficient, and humane methods for evaluating cosmetic ingredients and formulations.

### 11.2 Green and sustainable beauty

In today’s beauty landscape, an emerging and imperative theme is the pursuit of green and sustainable beauty practices (144). This transformative movement seeks to revolutionize industry by embracing environmentally conscious principles. One pivotal aspect of this shift revolves around eco-friendly packaging solutions. Brands are increasingly adopting sustainable packaging, opting for materials that are recyclable, biodegradable, and, in some instances, even reusable (145). This move not only reduces plastic waste but also aligns with the broader sustainability goals of minimizing the beauty industry’s ecological footprint.

Furthermore, the ingredients themselves are undergoing a fundamental transformation. Consumers are increasingly discerning, demanding products that contain natural, organic, and ethically sourced components (146). Brands are responding by formulating cosmetics with clean, green ingredients that not only enhance personal well-being but also minimize harm to the environment. This emphasis on ingredient sustainability is not merely a trend; it’s a fundamental shift in how cosmetics are conceived, developed, and marketed.

The commitment to sustainability extends beyond product formulation and packaging. Manufacturers are actively exploring ways to reduce their carbon footprint through energy-efficient production processes and the integration of renewable energy sources. This multifaceted approach reflects a growing awareness of the beauty industry’s environmental impact and the collective responsibility to reduce it (147).

Ethical considerations are at the forefront of green and sustainable beauty. Brands are taking steps to ensure that their products are cruelty-free, and they are working to create transparent and ethical supply chains (Gruber and Holweg, 2019). This means consumers can trust that the products they purchase not only align with their values but also promote responsible practices throughout the production chain (148).

Consumer empowerment plays a pivotal role in this paradigm shift. Certification programs, such as those for organic and cruelty-free products, enable shoppers to make informed choices. Simultaneously, consumer awareness campaigns are highlighting the importance of sustainable beauty practices and influencing purchasing decisions. Industry influencers and thought leaders are amplifying this message, further accelerating the demand for eco-conscious cosmetics (149).

As the regulatory landscape evolves and waste reduction initiatives gain momentum, the beauty industry is at a pivotal juncture. The green and sustainable beauty movement is not merely a fleeting trend but a fundamental reimagining of the industry’s future—one that prioritizes both personal well-being and the well-being of our planet.

### 11.3 Consumer empowerment in the beauty industry

In the modern beauty landscape, consumer empowerment stands as a formidable force that has reshaped the industry’s dynamics (150). Gone are the days when consumers were passive recipients of beauty standards and product offerings. Instead, today’s consumers are informed, discerning, and empowered individuals who are redefining beauty norms and expectations (151). This newfound consumer empowerment is driving transformative changes within the industry, influencing everything from product formulations to brand ethics. One of the key pillars of consumer empowerment in the beauty industry is the accessibility of information (152). With the advent of the internet and social media, consumers now have unparalleled access to a wealth of beauty-related information. They can research product ingredients, read extensive product reviews, and scrutinize brand practices in real-time. This shift has compelled beauty companies to be more transparent and accountable in their operations. Consumers can easily fact-check claims, pore over ingredient lists, and make choices that align with their individual needs and values. Clean and ethical beauty has surged in response to consumer empowerment (153). Consumers are increasingly concerned about the ingredients they apply to their skin and the impact of their beauty choices on the environment. As a result, clean beauty, characterized by formulations free from harmful chemicals, has gained traction. Additionally, consumers are seeking cruelty-free, vegan, and sustainably sourced cosmetics. Brands that prioritize these values are thriving in this consumer-driven landscape (154).

Certification programs have emerged as powerful tools of consumer empowerment (155). These programs, which include organic, cruelty-free, and sustainable certifications, empower consumers to identify products that meet specific standards and align with their principles. Labels such as “USDA Organic” or “Leaping Bunny Certified” provide consumers with a quick and reliable way to make choices that reflect their values. Influencer culture has played a pivotal role in amplifying consumer voices. Social media influencers and beauty bloggers wield significant influence in shaping trends and consumer preferences. Their reviews, recommendations, and critiques are trusted by millions, influencing purchasing decisions and holding brands accountable for product quality and ethical practices (156). Moreover, personalization has become a hallmark of consumer empowerment (157). Technology-driven innovations, such as AI-powered skincare analysis and custom cosmetics, enable consumers to tailor products to their unique needs. This level of customization empowers individuals to take control of their beauty routines, ensuring that products are not only effective but also perfectly suited to their specific skin types and concerns (158). Beyond individual choices, consumer empowerment has given rise to collective advocacy and activism (152). Consumers are increasingly vocal about issues such as diversity and inclusion, pushing brands to expand their shade ranges and represent a broader spectrum of beauty. Activism extends to sustainability and ethical practices, encouraging brands to reduce waste, support fair labor practices, and minimize their environmental impact. Lastly, the beauty industry’s accountability and responsiveness to consumer feedback have been transformed (150). Online platforms have democratized product reviews and brand accountability. Consumers can share their experiences and concerns with a global audience. Brands are compelled to respond to customer feedback, address issues promptly, and continuously improve their offerings to meet consumer expectations.

Overall, consumer empowerment in the beauty industry is a defining feature of the contemporary landscape. It places consumers at the center, giving them the tools, information, and platforms to shape the industry according to their values and preferences. As consumers continue to advocate for ethical, sustainable, and personalized beauty choices, the industry must respond with transparency, innovation, and a commitment to meeting the evolving needs of an empowered clientele.

## 12 Recommendations

The study of the harmful impacts of personal care and cosmetic products on human health highlights the need for increased knowledge, stricter laws, and wiser consumer decisions. As our knowledge of the complex connection between these goods and human health grows, it is crucial to take proactive steps to reduce potential hazards and create a safer environment for everyone (159). Governments, regulatory agencies, producers, and consumers must work together successfully to address this issue. Stricter laws must be upheld, with an emphasis on thorough ingredient vetting, exacting testing, and open labeling procedures. Based on advancing scientific understanding, regulatory bodies should continually review and update lists of drugs that are prohibited or restricted (160). The safety of the things they produce is crucially dependent on the manufacturers. The potential hazardous impact on human health will be greatly diminished by using cleaner and safer ingredient substitutes, investing in research and development for non-toxic formulations, and upholding ethical standards (119). Consumer education is also crucial. People can be empowered to make wise purchasing decisions if they are told about potentially harmful substances and given instructions on how to read product labels. Consumers will be able to recognize goods that support their ethical and physical well-being thanks to this awareness, which will increase demand for safer and more environmentally friendly items (61).

Furthermore, it is crucial to promote an accountable and transparent culture within the cosmetic and personal care sector. Encouraging businesses to reveal their testing procedures, sourcing methods, and sustainability programs (161, 162).

## 13 Conclusion

The varied appeal of beauty and personal care products undoubtedly boosts our confidence and aesthetic appeal, but there are unspoken consequences for human health. Several studies have shown alarming associations between the chemicals present in many of these products and a range of health problems, from minor skin irritations to more serious ailments like hormone imbalances, problems with reproduction, and even some types of cancer. Even though not all products are dangerous, our regular use of them means that we should be paying more attention to cumulative exposure to these poisons. Customers are frequently tricked into believing that product safety is strictly regulated. Regulating gaps, however, makes it possible for many potentially dangerous chemicals to be a part of our everyday lives. This necessitates a two-pronged response: consumers should educate themselves and look for natural and organic alternatives whenever feasible, and regulatory agencies should strengthen safety regulations. It’s critical to find equilibrium in the relationship between health and beauty so that pursuing exterior attractiveness does not compromise our internal well-being.

## Footnotes

^1^ https://eur-lex.europa.eu/legal-content/EN/TXT/HTML/?uri=CELEX:32021R1099&rid=1

^2^ https://single-market-economy.ec.europa.eu/sectors/cosmetics/cosmetic-ingredient-database_en

^3^ https://single-market-economy.ec.europa.eu/sectors/cosmetics/ban-animal-testing_en

^4^ https://single-market-economy.ec.europa.eu/sectors/cosmetics/cosmetic-products-specific-topics_en

^5^ https://eur-lex.europa.eu/legal-content/EN/TXT/?uri=CELEX:02009R1223-20190813

^6^ https://eur-lex.europa.eu/legal-content/EN/TXT/?uri=CELEX:02008R1272-20101201
